# *Lactobacillus johnsonii* N6.2 Mitigates the Development of Type 1 Diabetes in BB-DP Rats

**DOI:** 10.1371/journal.pone.0010507

**Published:** 2010-05-06

**Authors:** Ricardo Valladares, Dhyana Sankar, Nan Li, Emily Williams, Kin-Kwan Lai, Asmaa Sayed Abdelgeliel, Claudio F. Gonzalez, Clive H. Wasserfall, Joseph Larkin, Desmond Schatz, Mark A. Atkinson, Eric W. Triplett, Josef Neu, Graciela L. Lorca

**Affiliations:** 1 Department of Microbiology and Cell Science, University of Florida, Gainesville, Florida, United States of America; 2 Department of Pediatrics, University of Florida, Gainesville, Florida, United States of America; 3 Department of Pathology, Immunology, and Laboratory Medicine, University of Florida, Gainesville, Florida, United States of America; Louisiana State University, United States of America

## Abstract

**Background:**

The intestinal epithelium is a barrier that composes one of the most immunologically active surfaces of the body due to constant exposure to microorganisms as well as an infinite diversity of food antigens. Disruption of intestinal barrier function and aberrant mucosal immune activation have been implicated in a variety of diseases within and outside of the gastrointestinal tract. With this model in mind, recent studies have shown a link between diet, composition of intestinal microbiota, and type 1 diabetes pathogenesis. In the BioBreeding rat model of type 1 diabetes, comparison of the intestinal microbial composition of diabetes prone and diabetes resistant animals found *Lactobacillus* species were negatively correlated with type 1 diabetes development. Two species, *Lactobacillus johnsonii* and *L. reuteri,* were isolated from diabetes resistant rats. In this study diabetes prone rats were administered pure cultures of *L. johnsonii* or *L. reuteri* isolated from diabetes resistant rats to determine the effect on type 1 diabetes development.

**Methodology/Principal:**

Findings Results Rats administered *L. johnsonii,* but not *L. reuteri,* post-weaning developed type 1 diabetes at a protracted rate. Analysis of the intestinal ileum showed administration of *L. johnsonii* induced changes in the native microbiota, host mucosal proteins, and host oxidative stress response. A decreased oxidative intestinal environment was evidenced by decreased expression of several oxidative response proteins in the intestinal mucosa (Gpx1, GR, Cat). In *L. johnsonii* fed animals low levels of the pro-inflammatory cytokine IFNγ were correlated with low levels of iNOS and high levels of Cox2. The administration of *L. johnsonii* also resulted in higher levels of the tight junction protein claudin.

**Conclusions:**

It was determined that the administration of *L. johnsonii* isolated from BioBreeding diabetes resistant rats delays or inhibits the onset of type 1 diabetes in BioBreeding diabetes prone rats. Taken collectively, these data suggest that the gut and the gut microbiota are potential agents of influence in type 1 diabetes development. These data also support therapeutic efforts that seek to modify gut microbiota as a means to modulate development of this disorder.

## Introduction

More than 17 bacterial families encompassing 400 to 500 different microbial species can be found in human adults [1]. These commensal bacteria regulate a myriad of host processes and provide several nutrients to their host and their symbionts within the microbial community. In healthy individuals these relationships are thought to occur in equilibrium. However, disruption of this equilibrium may contribute to a variety of conditions including inflammatory bowel disease and atopy [2]. This connection is gaining credibility as associations between gut microbiota and either the risk for or presence of a variety of specific human diseases is demonstrated [2].

Genetics undoubtedly plays a major role in the development of type 1 diabetes (T1D), however numerous environmental factors have been suggested that could trigger genetic susceptibility. Interactions between the intestinal environment, barrier function, and immune system have been shown to have a major impact in the rate of T1D development. We previously proposed a hypothesis involving a trio of interacting factors that may create a “perfect storm” for T1D development [3]. These factors include (i) an aberrant intestinal microbiota [4], [5], (ii) a ‘leaky’ intestinal mucosal barrier [6], and (iii) altered intestinal immune responsiveness [7]. In support of this model, modulation of T1D pathogenesis in animal models has proved successful through early intervention with a variety of dietary alterations [8]. Indeed, the administration of a hydrolyzed casein diet [9] or the administration of antibiotics [4], [5] has strengthened the hypothesis that an aberrant microbiota could accelerate disease development. However, studies determining the effects of antibiotics in modulating disease development have not assessed whether reduction of what could be considered “pro-diabetogenic” flora, or alternatively, an overgrowth of protective flora occurs.

In the BioBreeding diabetes prone (BB-DP) model of T1D, rats spontaneously develop the automimmune disease due to genetic susceptibility. A previous study reported a culture-independent analysis of the bacteria in fecal samples collected from Biobreeding diabetes resistant (BB-DR) and BB-DP rats [10]. Two genera of bacteria, *Lactobacillus* and *Bifidobacterium,* emerged as dominant groups negatively correlated with the onset of T1D. Further analysis of the *Lactobacillus* population within these groups established that *Lactobacillus* strains with cinnamoyl esterase activity, *L. johnsonii* N6.2 and *L. reuteri* TD1, were negatively correlated with T1D development [11].

This report aims to establish whether commensal bacteria negatively correlated with T1D (i.e., present in BB-DR rats) can delay or prevent disease onset in BB-DP rats. As proof of concept, purified *L. johnsonii* isolated from BB-DR rats were fed to BB-DP rats. The resulting change in intestinal microbiota composition was tested for the ability to delay or prevent autoimmunity in BB-DP rats. A general assessment of the host response to *L. johnsonii* administration indicated that the microbe might target an early signaling pathway conducive to increased levels of interepithelial junction proteins and mucus secretion, while decreasing oxidative status and inflammation in the intestine.

## Results

### Decreased incidence of diabetes in BB-DP rats fed with *L. johnsonii* N6.2

**Figure 1:**
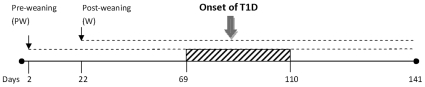
Feeding design using BB-DP animals. Arrows in black mark the time that feeding was started. The dashed line indicates daily feeding. The dashed box indicates the period in which rats developed T1D associated hyperglycemia.

**Figure 2:**
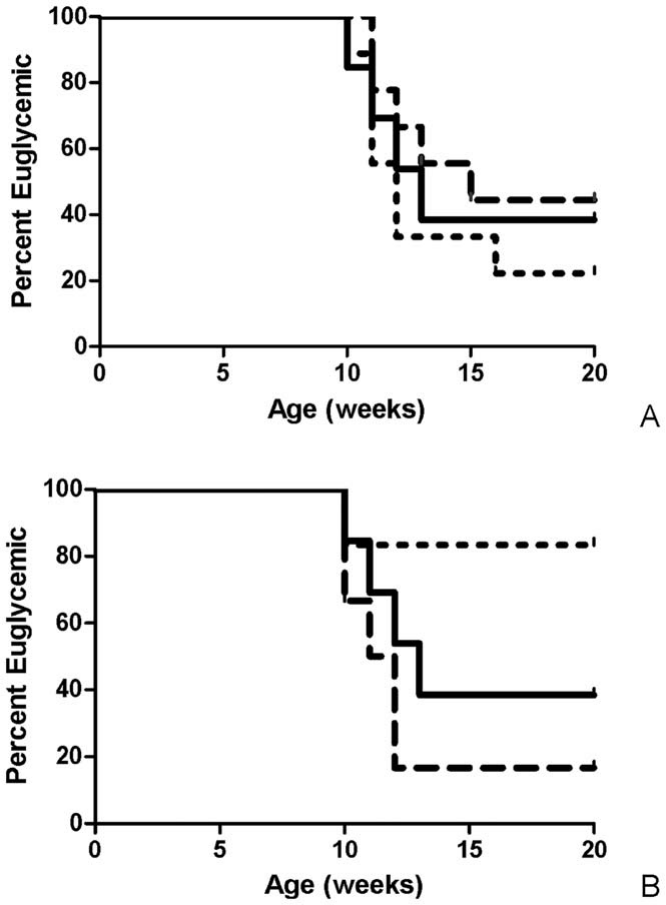
Kaplan-Meier plot depicting development of T1D in BB-DP rats. Rats fed A) pre-weaning or B) post-weaning with *L. johnsonii* N6.2 (short dashed line), or *L. reuteri* TD1 (long dashed line) compared to the PBS fed control (solid line) N = 10 per group.

*Lactobacillus* strains isolated from BB-DR rats were administered to BB-DP rats to analyze their effect on T1D development. *L. reuteri* TD1 or *L. johnsonii* N6.2 suspensions (1×10^8^ CFU per animal) were administered individually daily by oral gavage i) pre-weaning to 1 day old BB-DP rats during mother feeding and ii) post-weaning to 21 day old BB-DP rats (Fig. 1). The pre-weaning administration of *Lactobacillus* did not modify the rate of T1D development, however the post-weaning administration of *L. johnsonii* N6.2 decreased the incidence of T1D compared to the control animal group (BB-DP, N = 10, *P<*0.1). A more significant difference was observed when comparing the *L. johnsonii* N6.2 fed group to the *L. reuteri* TD1 fed group (*P*<0.04). While disease incidence in *L. johnsonii* fed animals decreased, the *L. reuteri* fed group showed a similar behavior as the control group (Fig. 2). The delay in T1D onset was specific to *L. johnsonii* fed animals. As a result, in the remainder of the study we compared the responses referring to three groups: (i) *L. johnsonii* fed group (includes only healthy animals), (ii) healthy controls, and (iii) diabetic animals (includes animals from both the diabetic control and *L. johnsonii* fed groups that developed T1D).

### Administration of *L. johnsonii* N6.2 modifies the intestinal microbiota

The impact of *L. johnsonii* N6.2 feeding on the intestinal microbiota was determined. Main groups of microorganisms were cultured at the onset of diabetes in sick animals or after 141 days in animals that remained healthy. The abundance of specific bacterial genera was also measured by RT-qPCR. No statistically significant differences were obtained in the stool culturable bacterial fractions of *Lactobacillus*, *Bacteroides*, or in the total anaerobe counts (data not shown). Interestingly, a close examination of the colony morphology on Rogosa plates showed a diversity of morphologies in the control group while the *L. johnsonii* and *L. reuteri* fed groups showed homogenous colony morphology. These observations were confirmed by isolation of bacteria (10 per plate per animal) and sequencing of the 16S RNA gene. In the control group, the most abundant specie was *L. murinus* (65% of isolates) while *L. intestinalis* (9%), *L. reuteri* (18%), *L. johnsonii* (8%) were found in lower proportions. In the *L. johnsonii* and *L. reuteri* fed groups 88% and 92%, respectively corresponded to the inoculated bacteria in each group while the remaining colonies were identified as *L. murinus*. Since these results indicate that predominant species of *Lactobacillus* are uniform within groups, observed differences in diabetes development and intestinal environment between groups may correlate to observed differences in intestinal microbiota composition.

**Figure 3:**
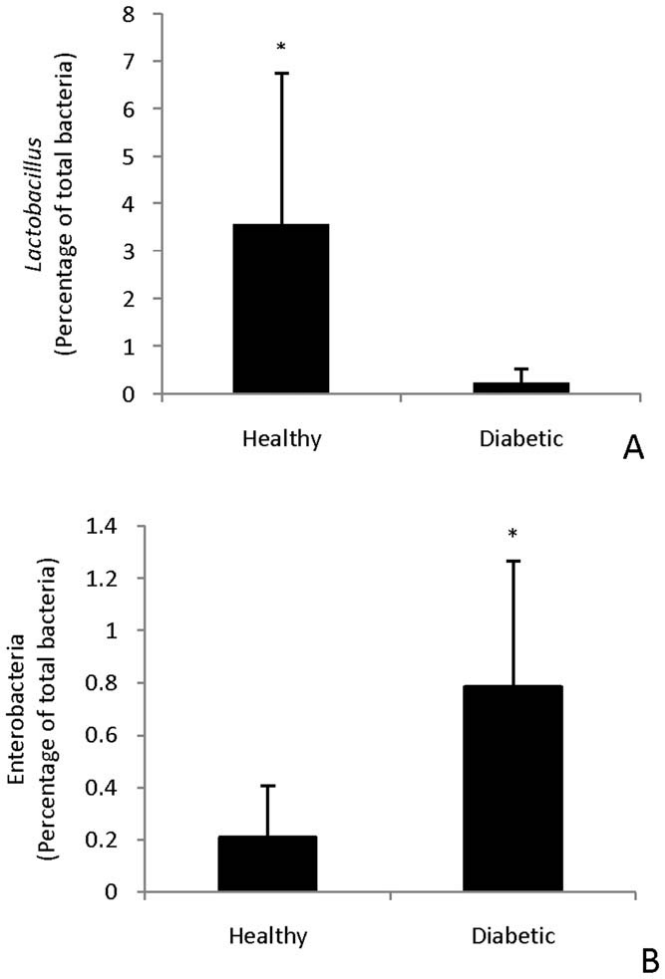
Quantification using real time qPCR of lactobacilli (A) and enterobacteria (B) from ileal mucosa. The values are expressed as mean of the percentages from total bacteria determined from 5 ng of DNA. * indicates significant differences (*P*<0.05) between healthy and diabetic animals (N = 6 per group).

Similarly, no significant differences were obtained in RT-qPCR experiments measuring the concentration of *Pseudomonas, Bacteroides, Staphylococcus, Bifidobacterium, Clostridium, Lactobacillus,* and enterobacteria in the ileal and colonic content. However, analysis of the ileal mucosa unveiled a statistically significant increase in the *Lactobacillus* population in all rats that did not developed diabetes, independent of bacterial administration. On the other hand, a statistically significant increase in concentration of enterobacteria was found in all diabetic animals, independent of bacterial administration (Fig. 3). Since no differences in the microbiota were obtained in the stool samples, but were statistically significant in the ileal mucosa, the positive effect of *L. johnsonii* N6.2 could be exhibited primarily in the intestinal mucosa.

### *L. johnsonii* N6.2 administration modifies expression of tight junction proteins

**Figure 4:**
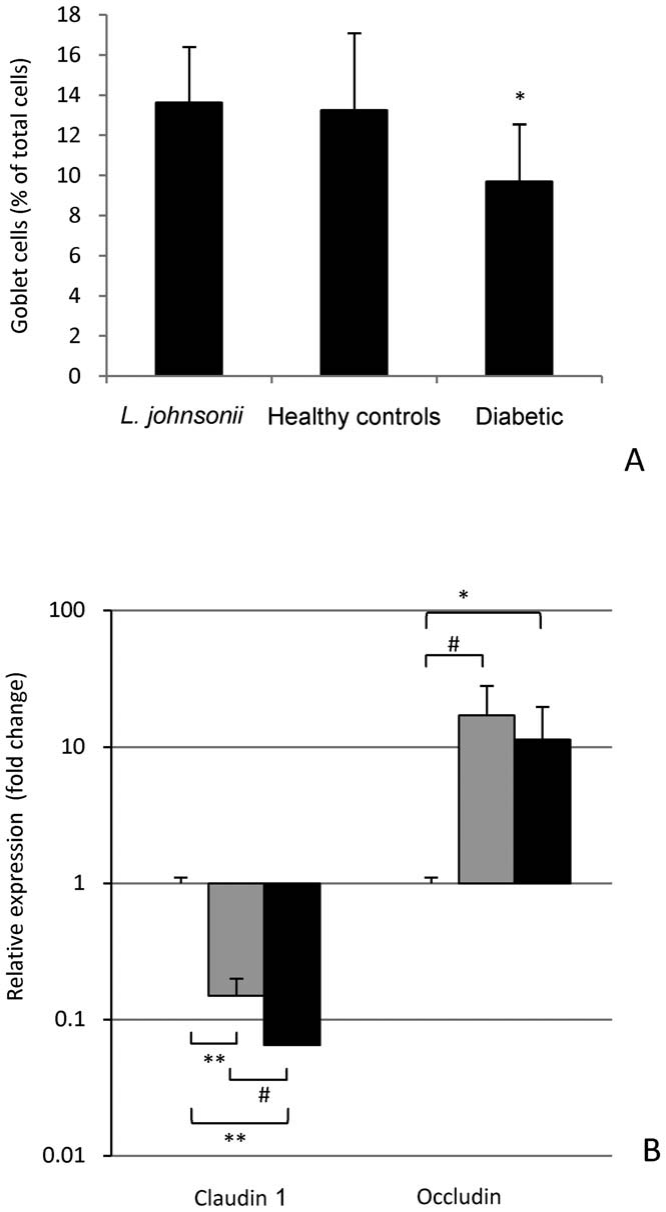
Effect of L. johnsonii administered post weaning on prevalence of goblet cells (A) and on mRNA levels of tight junction genes (B). Hematoxylin and eosin stained slides of distal small intestine were examined for morphological changes. (A) Percentage of goblet cells in the distal small intestine by treatment group. (B) RT-qPCR analysis of the expression of tight junction genes. Relative amounts of claudin 1 and occludin were calculated by subtracting the internal control (β actin) and changes in expression levels were calculated relative to the value in the *L. johnsonii* fed group (expression  = 1). Grey bars: Relative expression in the healthy control, Black bars: relative expression in the diabetic animals. The values are means +S.D. (N = 6); * *P*<0.05; ^#^ *P*<0.01; **P<0.0001

Previous studies have reported lower levels of the major intercellular tight junction protein claudin-1 and greater intestinal permeability in the BB animal model before the onset of diabetes [6]. It has been suggested that early increase in intestinal permeability in the BB-DP rats may allow unregulated passage of environmental antigens that could trigger the autoimmune response culminating in T1D development. To determine if administration of *L. johnsonii* N6.2 modified intestinal integrity, macroscopic modifications in the mucosal architecture were examined on hematoxylin and eosin stained slides of distal small intestine. No morphological differences between the *L. johnsonii* fed group, control healthy, or diabetic animals were found in villus height or width or crypt depth (data not shown). Necrosis was not observed in any samples. Interestingly, the number of goblet cells was significantly higher in both healthy controls and *L. johnsonii* fed animals when compared to the diabetic group (Fig. 4A, *P*<0.05).

At the molecular level we measured the expression of genes encoding claudin-1 and occludin proteins involved in intercellular tight junction assembly and maintenance in the intestine. The healthy control and diabetic groups showed low expression of the sealing claudin-1 and high levels of occludin tight junction related transmembrane protein compared to the *L. johnsonii* fed group (Fig. 4B). Specifically, the feeding of *L. johnsonii* upregulated the expression of claudin-1 and decreased the expression of occludin. Small differences were observed when comparing the healthy control group to the diabetic animals. Consequently, *L. johnsonii* N6.2 feeding could ameliorate the intestinal barrier dysfunction observed in this animal model.

### *L. johnsonii* decreases the host intestinal oxidative stress response

Among the destructive effects of reactive oxygen species (ROS) generated during early intestinal disease development is the disruption of epithelial tight junctions [12]. The levels of hexanoyl-lysine, a biomarker for oxidative stress [13], were determined by ELISA on ileal mucosa and were variable among the animals tested. However, levels were significantly higher (*P*<0.05) in diabetic animals (53±21 µM min^−1^) when compared to *L. johnsonii* group and healthy controls (14±10 µM min^−1^).

**Figure 5:**
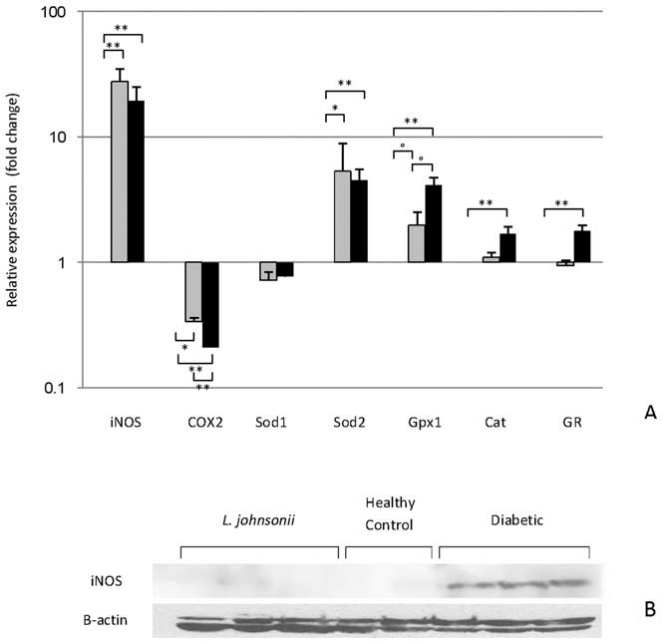
Assessment of the oxidative stress response in the host. (A) RT-qPCR analysis of the expression of genes linked to the oxidative stress response in the host. Relative amounts of iNOS, Cox2, Sod1, Sod2, Gpx1, Cat, and GR were calculated by subtracting the internal control (β actin) and changes in expression levels were calculated relative to the value in *L. johnsonii* feed group (expression  = 1). Grey bars: relative expression in the healthy control; Black bars: relative expression in the diabetic animals. The values are means +S.D. (N = 6); *P<0.05; °*P*<0.01, **P<0.0001. (B) Western blot analysis of iNOS levels. β-actin was used as internal control.

To determine the specific mechanisms involved, the ileal mRNA levels of enzymes involved in ROS detoxification pathways in the host were determined (Fig. 5A). To ease the presentation of data we illustrated the gene levels in terms of expression in the *L. johnsonii* fed group. The value of one represented expression levels in the *L. johnsonii* fed group. Of the genes measured, superoxide dismutase 2 (Sod2), catalase (Cat), glutathione reductase (GR), and glutathione peroxidase (Gpx1) were induced in diabetic animals compared the *L. johnsonii* group. Sod2 and Gpx1 were induced in the healthy controls compared the *L. johnsonii* group. Superoxide dismutase 1 (Sod1) was the exception as it was not modified under any condition. The expression of Sod2 and Gpx1 was induced in the diabetic animals (∼4.5 fold and ∼4 fold, respectively; *P*<0.05) and to a lesser extent Cat and GR (∼2 and 1.8 fold, respectively). By comparing the mRNA levels of the healthy (control and *L. johnsonii* fed) with diabetic animals two primary responses were observed: (i) genes negatively correlated with healthy status (Cat and GR), and (ii) genes negatively correlated with *L*. *johnsonii* administration (Sod2 and Gpx1).

ROS lead to the synthesis of nitric oxide by inducible nitric oxide synthase (iNOS). The mRNA levels of iNOS were significantly repressed in the *L. johnsonii* fed group when compared to diabetic animals as well as healthy controls (∼22 fold, Fig. 5A, *P*<0.0001). However, western blot analysis showed that iNOS is reduced in both *L. johnsonii* fed and healthy control groups (Fig. 5B) indicating that low levels of iNOS are correlated with the healthy status of the animals. Repressed expression of Cox2 in the diabetic group (Fig. 5A, *P*<0.001) and healthy control group in comparison to the *L. johnsonii* group was also observed.

**Figure 6:**
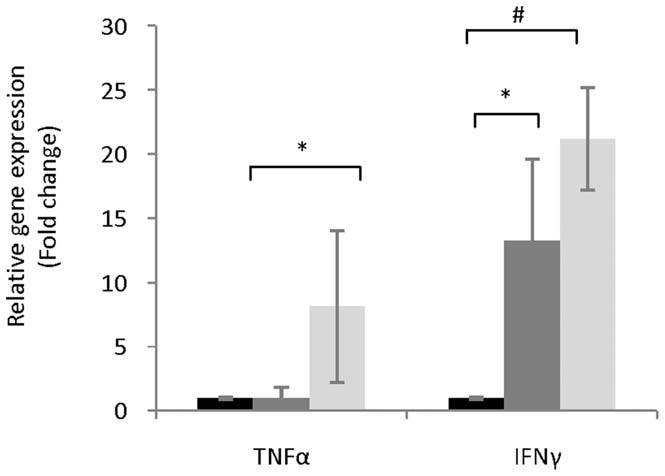
mRNA levels of the pro-inflammatory cytokine genes, IFNγ and TNFα linked to the oxidative stress response in the host. Relative expression was calculated as previously described relative to the value in the *L. johnsonii* feed group (expression  = 1). Relative expression in the *L. johnsonii* feed group (black bars), healthy control (dark grey bars), and diabetic animals (grey bars). The values are means +S.D. (N = 6); *P<0.05; ^#^ *P*<0.01.

IFNγ is an important mediator of inflammatory responses with pleiotropic effects in the host. It was previously reported that IFNγ induces the expression of iNOS [14] while repressing the expression of Cox2 [15]. The aim was to determine if a negative correlation existed between the levels of pro-inflammatory cytokines, particularly IFNγ and TNFα, and the *L. johnsonii*-mediated decrease in oxidative stress response in the host. The mRNA levels of TNFα decreased ∼7 fold (*P*<0.05) between the healthy and diabetic animals, but no differences between the healthy control group and *L. johnsonii* fed group were observed (Fig. 6). The results indicate that the low expression of TNFα is correlated with healthy status and not with the administration of bacteria. The expression of IFNγ, on the contrary, was directly linked to the administration of *L. johnsonii*. Diabetic animals exhibited a ∼20 fold higher expression (*P*<0.005) of IFNγ compared to the *L. johnsonii* fed group. No significant differences were observed between the healthy controls and diabetic animals indicating a specific contribution of the probiotic microorganism to the decrement of the inflammatory response.

## Discussion

In this study we report that the administration of *L. johnsonii* N6.2 decreased the progression to T1D when administered post-weaning. Among the possible mechanisms in which *Lactobacillus* exerts beneficial effects for the host are: (i) as a physical barrier inhibiting the passage of inflammatory antigens, (ii) degradation of toxic components, (iii) release of nutrients, and (iv) production of anti-inflammatory compounds. In the BB rat model a combination of all of these mechanisms is possible due to the overly permeable characteristic of the small intestine [6].

A higher number of goblet cells in the *L. johnsonii* fed and healthy control groups was observed when compared to the diabetic group. Goblet cells constantly produce mucus, which has a dual role of protecting the mucosa from adhesion of certain microorganisms while providing an initial binding site, nutrient source, and matrix on which commensal bacteria can proliferate [16]. In this study the observed decrease in goblet cells correlated with the sick status of the animals, which could result in a lower production of intestinal mucins. Of note, Mack *et al*. [17] showed that *L. plantarum* has a direct effect on intestinal epithelial cells by inducing secretion of mucins that diminish enteric pathogens binding to mucosal epithelial cells. The sustained mucus production present in *L. johnsonii* and healthy control groups could prevent damage from enteric pathogens. While a higher number of goblet cells was associated with all healthy specimens, claudin expression was specifically induced following feeding of *L. johnsonii.* This change in expression indicates a direct effect of the bacteria on intestinal barrier integrity.

A puzzling result was that the T1D preventative effect of *L. johnsonii* was only observed when administration began in the post-weaning period rather than during pre-weaning. A combination of scenarios could explain these results. Since the pre-weaning group received continued *L. johnsonii* administration throughout the experiment (Fig.1), the beneficial effects of these bacteria on T1D development may be dependent on host immune system maturity. It is possible that the stress generated by very early intervention in specimen development could offset the balance of microbial intestinal composition or immune responsiveness.

The difference in prevention capabilities between *L. reuteri* and *L. johnsonii* post-weaning may involve their response to diet composition as this factor has been shown to be an important contributor to development of T1D [18], [19]. Although the effects of specific diets have been reported, the mechanisms behind these effects remain obscure. One possibility is that certain commensal bacteria such as *L. johnsonii* exert beneficial effects on the host intestines by releasing antioxidant compounds through dietary fiber hydrolysis. The release of antioxidant compounds by probiotic bacteria is relevant since an enhanced oxidative stress response triggered by the excessive production of reactive oxygen species is observed in T1D and other diseases [20], [21]. It has been shown that small doses of antioxidant compounds decrease the incidence of diabetes in streptozotocin (STZ)-induced diabetic mice [22]. Also, we have previously reported a negative correlation between the presence of bacteria able to release bioactive antioxidant components from phenolic compounds and the BBDP model [11]. The *L. johnsonii* N6.2 strain used in this study possesses two esterases that can release cinnamic acid and other phenolic compounds with anti-inflammatory properties. However, the direct role of these enzymes and antioxidant compounds on T1D pathogenesis requires further investigation.

The oxidative status of the ileal mucosa was assessed by measuring the mRNA levels of genes involved in the host oxidative stress response. Compared to the *L. johnsonii* fed group, genes encoding Sod2, Gpx1, Cat, and GR were induced in diabetic animals. However, Gpx1 and Sod2 expression was also induced in healthy controls compared to the *L. johnsonii* group. Overall, the lower level of these markers of oxidative stress in the *L. johnsonii* group indicates a more favorable anti-inflammatory environment in the ileum with lower levels of ROS.

Nitric oxide is a signaling molecule that links inflammation and the development of T1D. An increased transcription and translation of the inducible nitric oxide synthase (iNOS) gene has been associated with T1D development in BB-DP rats [23], [24]. Here, the expression level of iNOS (and its inducing cytokine, IFNγ) was downregulated in the *L. johnsonii* group compared to the diabetic control group. Interestingly, the levels of Cox-2 showed the opposite effect. Cox-2 has been reported to be mainly induced in activated macrophages and other inflammatory cells [25]. However, Luo *et al*. [26] showed that the presence of Cox-2 in β-cells decreased during progression of diabetes in the NOD mouse model. In this study, the mRNA levels of Cox-2 in the ileum increased in the healthy animals with the highest expression in the *L. johnsonii* fed group. The increase in Cox-2 expression observed also correlates with a higher number of goblet cells in the intestine of healthy rats, in agreement with Luo *et al.* [27]. However, these results suggest that the expression of Cox-2, and its prostaglandin products, may have a protective effect. Gilroy, Colville-Nash and others [28], [29] have shown that the synthesis of cyclopentenone prostaglandins are determinant during inflammatory resolution.

In this study, low expression of pro-inflammatory cytokines IFNγ correlated with the administration of *L. johnsonii* N6.2, whereas low expression of TNFα correlated with overall healthy status. These results indicate that the mechanisms involved in T1D inhibition in this study may be different from the effect mediated by the general probiotic treatment VSL3. Previous reports in the NOD model of diabetes have shown that the administration of the probiotic formulation VSL3 decreased the incidence of T1D through IL10 immunomodulation [30] and induction of pro-inflammatory cytokine IFNγ.

Is has been suggested that a highly permeable epithelium, or ‘leaky gut’, fails to inhibit the passage of intestinal content antigens. This inappropriate exposure to antigens may trigger regulatory T-cells in an autoimmune cascade [3]. We propose that presence of *L. johnsonii* N6.2 in the intestinal epithelium helps maintain epithelial barrier function by increasing mucus and tight junction protein production while decreasing the effects of ROS. *L. johnsonii* may be targeting an early step in the inflammatory signaling pathway resulting in a more tolerogenic environment which reduces the overall oxidative stress.

In summary, this study observed a delay in the onset of T1D as well as physiological and immunological differences in the gut which correlated with the presence of *Lactobacillus johnsonii* N6.2. Nevertheless, further research is needed to elucidate the intricacies of the relationship between autoimmune disease, intestinal health, and gut microbiota.

## Materials and Methods

### Ethics Statement

All animal work has been approved by the University of Florida Institutional Review Board (IRB). Animal housing standards were as prescribed by the Association for Assessment and Accreditation of Laboratory Animal Care (AAALAC) with two male or female rats per cage under pathogen-free conditions. All rats were in the same room at the same temperature and under the same light. All rats received the same amount of water and food. The weights of these animals were measured weekly. The analysis indicated that all animals were gaining weight at the same pace with no significant discrepancies when healthy.

### Bacterial strains

Two bacterial strains: *Lactobacillus johnsonii* N6.2 or *Lactobacillus reuteri* TD1 isolated from BB-DR rats [11] were grown in MRS broth (REMEL, Lenexa, USA) at 37°C for 16 h. Cells were centrifuged at 3000 rpm, pellets washed with sterile PBS buffer. Aliquots containing 10^10^ cells ml^−1^ were stored at −80°C until used. Cell viability was determined by plate dilution method on three aliquots after thawing. For feeding experiments, new aliquots were thawed immediately before administration.

### BB-DP rats experimental design

Bacterial strains were administered to BB-DP rats (Biomedical Research Models, Worcester, MA) to test whether they would delay or inhibit the onset of T1D. *L. reuteri* TD1 or *L. johnsonii* N6.2 suspensions (10^8^ CFU) were administered daily by oral gavage i) pre-weaning to 1 day old BB-DP rats during mother feeding and ii) post-weaning at 21 days old BB-DP rats. The control group was administered PBS only. Starting at the age of 60 days, the blood glucose levels of the animals were taken weekly using a glucose monitor kit. If the glucose levels were higher than 250 mg/dl for two consecutive days, then the rat was considered to have developed diabetes. Once a rat developed diabetes, it was sacrificed, and organs and tissues were harvested and preserved for analysis. For anatomic studies, the small intestine was harvested and flushed with phosphate-buffered saline at 4°C to remove intraluminal contents. The small intestine was divided into 3 equal sections to demarcate the duodenum, jejunum and ileum. Small pieces of each section were fixed in 10% (v/v) neutral buffered formalin for 24 hours for light microscopy or preserved in RNAlater® solution (Ambion, Austin, USA) for RT-qPCR analysis.

### Analysis of the intestinal microflora by viability counts

Samples taken from colonic content were immediately placed in 5 ml of sterile PBS buffer. After dilution viable counts were obtained using MRS (REMEL, Lenexa, USA) adjusted to pH 5.5 for lactobacilli, BBE (BD BBL, Sparks, USA) for Bacteroides and BHI agar with sheep blood (REMEL, Lenexa, USA) for anaerobes incubated anaerobically (BD BBL™ GasPack Plus; Sparks, USA) for 48 h at 37°C. Enterobacteria counts were determined using McConkey agar plates (REMEL, Lenexa, USA) incubated under aerobic conditions at 37°C for 24 h.

### Analysis of the intestinal microflora by Real-time quantification

DNA extractions from samples preserved at −80°C in RNAlater® solution (Ambion, Austin, USA) were perform using the QIAamp DNA Stool Mini kit (Qiagen Sciences, Maryland, USA) following the manufacturer's instructions. We investigated the most important groups of the rat fecal microbiota using DNA extracts from each rat as a template for RT-qPCRs using the primers described on Table 1. Quantitative PCRs were performed in a reaction volume of 20 µl containing 1x iQ SYBR Green Supermix (Bio-Rad, Hercules, USA), 200 nM each forward and reverse primers, and 5 ng of DNA extracted from the stool samples. DNA concentrations were determined with the Nanodrop™ spectrophotometer. Amplification and detection of DNA were performed in duplicate with the iCycler detection system (BioRad, Hercules, USA) with optical grade 96-well PCR plates and optical film. The reaction conditions were 50°C for 2 min and 95°C for 10 min, followed by 45 cycles of 95°C for 15 s and 62°C for 1 min. Data analysis was conducted with the software supplied by Bio-Rad (Hercules, USA).

**Table 1:** Primer sequences based on the 16S rRNA used to discriminate between different groups of bacteria by RT-qPCR.

TARGET	NAME	SEQUENCE	SOURCE
Bacteria	F_Bact 1369	CGGTGAATACGTTCCCGG	[34]
	R_Prok 1492	TACGGCTACCTTGTTACGACTT	
*Lactobacillus*	F-lacto	GAGGCAGCAGTAGGGAATCTTC	[35]
	R-lacto	GGCCAGTTACTACCTCTATCCTTCTTC	
*Bacteroides*	AllBac296F	GAGAGGAAGGTCCCCCAC	[36]
	AllBac412R	CGCTACTTGGCTGGTTCAG	
*Clostridium*	Ccoc 07	GACGCCGCGTGAAGGA	[34]
	Ccoc 14	AGCCCCAGCCTTTCACATC	
Enterobacteriaceae	En-lsu-3F	TGCCGTAACTTCGGGAGAAGGCA	[37]
	En-lsu-3R	TCAAGGACCAGTGTTCAGTGTC	
*Pseudomonas*	PSD7F	CAAAACTACTGAGCTAGAGTACG	[37]
	PSD7R	TAAGATCTCAAGGATCCCAACGGCT	
*Staphylococcus*	STPYF	ACGGTCTTGCTGTCACTTATA	[37]
	STPYR2	TACACATATGTTCTTCCCTAATAA	
*Bifidobacterium*	F-bifido	CGCGTCYGGTGTGAAAG	[35]
	R-bifido	CCCCACATCCAGCATCCA	

DNA amplification standard curves were constructed using purified genomic DNA in the range 10 fg to 1 ng of *L. reuteri, L. johnsonii, Staphylococcus sp., Bacteroides dorei and E. coli* as previously described in Roesch et al. [10]. The conversion of the amount DNA of the different bacterial groups into cell numbers in the stool samples was determined considering the genome size for each bacteria and the copy number of the 16S RNA gene as described by Byun et al. [31] and Matsuda et al. [32].

### Intestinal morphology

Intestinal integrity was evaluated by histology. Neutral buffered formalin (10%, V/V) -fixed ileum samples were embedded in paraffin; cut into 4 µm sections, mounted on glass slides, and stained with hematoxylin and eosin (H&E) according to standard procedures. Villus height, width and crypt depth were measured using a Nikon microscope (Universal Imaging Corp., Westchester, PA) with an ocular micrometer without the examiner knowing the group assignment. The intestinal injury was evaluated using a semiquantitative scoring system ranging from 0 to 4 modified by Arumugam et al. [33]. Normal mucosa was scored as grade 0. Epithelial cell damages, including loss of cells and separation of the epithelial cells from the underlying villus were scored between grades 1–3, while loss of villus tissue was scored as grade 4. Intestinal sections were also analyzed for goblet cells per total cells within a villus. For each animal, counts from 6 villi for each slide in three different regions of the slide were averaged.

### Real-Time qPCR of host responses

DNA and RNA extractions from samples preserved at −80°C in RNAlater® solution (Ambion, Austin, USA) were perform using the Ilustra™ TriplePrep kit (GE Health care, UK) following the manufacturer's instructions. cDNA was synthesized using iScriptTM cDNA synthesis kit (Bio-Rad, Hercules, USA) and qRT-PCR were performed as described above. The primers used are described in Table 2.

**Table 2:** Primer sequences used to analyze the host response by RT-qPCR.

TARGET	NAME	SEQUENCE	SOURCE
β-actin	β-actin Fw	TGACAGGTGCAGAAGGAGA	[38]
	β-actin Rv	TAGAGCCACCAATCCACACA	
Claudin-1	Cldn-1_Fw	AGGTCTGGCGACATTAGTGG	[39]
	Cldn-1_Rv	TGGTGTTGGGTAAGAGGTTG	
Occludin	Occludin_Fw	GCTCAGGGAATATCCACCTATCA	[40]
	Occludin_Rv	CACAAAGTTTTAACTTCCCAGACG	
Interferon-γ	IFNγ_Fw	AGGATGCATTCATGAGCATCGCC	[41]
	IFNγ_Rv	CACCGACTCCTTTTCCGCTTCCT	
Tumor Necrosis Factor-α	TNF-a_Fw	TCTTCTCATTCCTGCTCGTG	[42]
	TNF-a_Rv	GATGAGAGGGAGCCCATTT	
Inducible Nitric Oxide Synthase	iNOS_Fw	CTCACTGTGGCTGTGGTCACCTA	[38]
	iNOS_Rv	GGGTCTTCGGGCTTCAGGTTA	
Glutathione Peroxidase 1	GPX1_Fw	CGGTTTCCCGTGCAATCAGT	[43]
	GPX1_Rv	ACACCGGGGACCAAATGATG	
Catalase	CAT_Fw	CGACCGAGGGATTCCAGATG	[43]
	CAT_Rv	ATCCGGGTCTTCCTGTGCAA	
Glutathione Reductase	GR_Fw	AGCCCACAGCGGAAGTCAAC	[43]
	GR_Rv	CAATGTAACCGGCACCCACA	
Superoxide Dismutase1	SOD1_Fw	GCGGTGAACCAGTTGTGGTG	[43]
	SOD1_Rv	AGCCACATTGCCCAGGTCTC	
Superoxide Dismutase 2	SOD2_Fw	AGCTGCACCACAGCAAGCAC	[43]
	SOD2_Rv	TCCACCACCCTTAGGGCTCA	
Cyclooxygenase-2	COX2_Fw	CTCTGCGATGCTCTTCCGAG	[44]
	COX2_Rv	AAGGATTTGCTGCATGGCTG	

### Western Blot analysis of iNOS expression

Protein expression was analyzed using whole cell lysates. Rat ileum samples were weighed, minced, and disaggregated by incubation at 250 rpm and 37°C in phosphate-buffered saline (1:2, w/v) with 0.25% collagenase. Samples were immediately place on ice and homogenized by vortexing with glass beads (Sigma Life Science) containing Complete Mini Protease Inhibitor Cocktail (Roche, Mannheim Germany). Samples were centrifuged at 13,000×g for 10 min at 4°C. Protein concentration was determined by Bradford method (Bio-Rad Protein Assay). 40 ug of protein per sample was separated using sodium dodecyl sulfate-polyacrylamide electrophoresis and transferred onto Nitroplus membranes (MSI, Flanders Ma) using a semi-dry transfer method. Membranes were blocked for 1 h in phosphate buffered saline with 0.075% tween 20 (T-PBS) and 5% milk. Membranes were incubated with mouse anti-iNOS antibody (1∶1000) (Abcam, Cambridge, Ma) or mouse anti- β-actin (1∶10,000) (Abcam, Cambridge, Ma) in T-PBS at 4°C overnight, and subsequently washed twice in T-PBS for five minutes. Incubation in horseradish peroxidase-conjugated anti-mouse antibody was performed for 1 h and signal was detected using enhanced chemiluminescent system (Amersham Pharmacia Biotech). β-actin was utilized as an internal control.

### Hexanoyl-lys enzyme-linked immunosorbent assay

Relative lipid peroxidation was determined by analyzing hexanoyl-lys levels by ELISA on rat ileum cell suspensions. 20 mg of each rat ileum sample was finely minced on a cold glass slide and suspended in 300 ul 1X PBS +0.25% collagenase type I (Invitrogen, Carlsbad, California). Samples were vortexed vigorously at 5 min intervals while incubating at 37°C for 30 min. Free cell suspensions were separated from remaining connective tissue fractions after incubation. Cell concentration was determined by optical density at 600 nm using a Syngery HT microplate reader (BioTek Instruments, Winooski, VT) Each sample was split into 1 ml aliquots (control and experimental sets), pelleted at 5000 rpm, and washed with 1 ml 1X PBS twice. Cell pellets were resuspended in 100 µl of 1X PBS and 100 µl of 1X PBS was added to the control set, and 100 µl of 1X PBS +2 µg/ml hexanoyl-lysine monoclonal antibody (JaICA, Shizuoka, Japan) was added to the experimental set. Samples were incubated at 37°C for 1 hr, followed by two washes in 1X PBS. 100 ul of 1X PBS +80 ng/ml of peroxidase labeled anti-mouse monoclonal antibody (Amersham Pharmacia Biotech, Pittsburgh, PA) was added to both sets and incubated for 1 hr at 37°C. Following incubation, cells were washed twice and resuspended in 100 ul 1X PBS. A 20 mM reaction mixture of o-Phenylenediamine (Sigma, St. Loius, MO) was prepared in a 50 mM phosphate citrate buffer pH = 5 and kept dark. Immediately before reading, 30% H_2_O_2_ was added to the reaction mixture for a final concentration of 0.04%. 100 ul of reaction mixture was added to each sample, and samples were read continuously at 450 nm and 570 nm for 30 min using Syngery HT microplate reader (BioTek Instruments, Winooski, VT). Specific activity was calculated as the amount of product (µmol.min^−1^) and normalized the number of cells. This assay was performed in triplicate for each sample.

### Statistical Analysis

Statistical analysis for significant differences was performed according to the Student's *t*-test for unpaired data or by the nonparametric Mann–Whitney. Differences with *P*<0.05 and lower were considered significant. Data was analyzed by GraphPad Prism (GraphPad Software, San Diego, USA).
